# Sericin Ameliorates the Capacitation State and Chromatin Integrity of Frozen-Thawed Stallion Spermatozoa by Reducing Oxidative Stress

**Published:** 2019

**Authors:** Mahboobeh Heidari Nasirabadi, Abolfazl Shirazi, Ali Kadivar, Naser Shams-Esfandabadi, Abdolnaser Mohebbi, Ebrahim Ahmadi

**Affiliations:** 1. Department of Clinical Sciences, Faculty of Veterinary Medicine, Shahrekord University, Shahrekord, Iran; 2. Research Institute of Animal Embryo Technology, Shahrekord University, Shahrekord, Iran; 3. Reproductive Biotechnology Research Center, Avicenna Research Institute, ACECR, Tehran, Iran

**Keywords:** Antioxidants, Glutathione, Sericins, Spermatozoa

## Abstract

**Background:**

In the process of sperm cryopreservation, apart from cryoinjury, the production of Reactive Oxygen Species (ROS) can adversely affect the integrity of chromatin and cellular membranes. Addition of natural antioxidants to freezing medium is an approach to reduce the destructive effects of ROS on sperm.

**Methods:**

In this study, during 60 *min* of cooling process, the ejaculates of five stallions were diluted in the following media: INRA 82 medium as Control (C), INRA 82 medium supplemented with 0.25% Sericin (S), INRA 82 medium supplemented with 1.5 *mM* Glutathione (G), and INRA 82 medium supplemented with 0.25% Sericin+1.5 *mM* Glutathione (S+G).

**Results:**

In the frozen/thawed sericin supplemented group, while the integrity of DNA and the activity of catalase and Glutathione Peroxidase (GPx) were increased, the lipid peroxidation and midpieceab normality decreased, compared with other groups (p<0.05). The proportions of sperms with abnormal head in group S and the sperm with distal droplet in G and S+G groups decreased, compared with group C (p<0.05). In CTC assay, the percentage of capacitated spermatozoa in treatment groups was lower than control (p<0.01).

**Conclusion:**

In conclusion, the presence of sericin in freezing medium of stallion semen could improve sperm DNA integrity and its resistance to ROS and lipid peroxidation.

## Introduction

Despite the successes achieved in semen cryopreservation in the cattle industry, there are some limitations in generalization of this technology in the equine breeding industry 1. One of the most important of these limitations is the destructive effect of freeze/thaw process on sperm quality arising from physical and chemical stresses on the sperm plasma membrane. Part of these destructive effects is associated with oxidative stress due to production of Reactive Oxygen Species (ROS) 2. In equine, Polyunsaturated Fatty Acids (PUFAs) of sperm plasma membrane and its DNA structure are specifically vulnerable to oxidative stress 3.

Sperm is protected against ROS and Lipid Peroxidation (LPO) through two principal strategies, using enzymatic and non-enzymatic antioxidants. Glutathione Peroxidase (GPx), Superoxide Dismutase (SOD), and Catalase (CAT) are the most critical enzymatic antioxidants 4,5 and reduced glutathione (GSH), α-tocopherol, ascorbic acid, carotenoids, ubiquinones, taurine, and hypotaurineserve as non-enzymatic antioxidants 6,7. Any disproportion between the antioxidant systems and ROS contents due to seminal plasma dilution during semen processing and/or during freeze-thaw process leads to a significant reduction in the defense system against free radicals and increases the destructive effects of LPO 3. To overcome this problem, various antioxidants were added to semen extenders of different species. A considerable body of evidence indicates that extender supplementation with antioxidants before cryo-preservation has improved the post-thaw motility, membrane integrity, and fertilizing ability of sperm infeline 8, canine 9, ovine 10, caprine 11, bovine 12, buffalo 13 and equine 14 species.

Glutathione (L-γ-glutamyl-L-cysteinylglycine, GSH) is a tripeptideubiquitously found in mammalian spermatozoa and seminal plasma 15 which with thiol (SH) groups has antioxidant characteristics. Therefore, it has an important role to directly react with ROS and work as a co-factor for GPx to reduce both hydrogen peroxide to H_2_O and lipoperoxides to alkyl alcohols 16. Based on evidence, the glutathione concentration of semen significantly depletes throughout storage period 17,18. Additionally, it has been demonstrated that supplementation of extender with low dosage of glutathione improves the spermatozoa quality in some species 17,19,20.

Sericin as a water soluble component of silk which contributes to 20–30% of the total cocoon weight, has an antibacterial and UV resistant properties 21,22. Its molecular weight ranges from 10 to 310 *kDa*, containing 18 kinds of amino acids 23 with high content of the hydroxyl groups of hydroxyamino acids (Serine and threonine) which can suppress *in vitro* lipid peroxidation and tyrosinase activity 24. It is indicated that culture medium supplementation with 0.5% sericin improves the developmental competence of bovine embryo by protecting against oxidative stress 25. It has been also demonstrated that the addition of sericin to freezing medium of various mammalian cells is comparable to the conventional medium containing serum 26. Sericin as a cell membrane-impermeable cryoprotectant with inhibitory effect against ice crystal formation may protect the cells from cryodamage 27. However, no information is available related to the addition of sericin to the freezing medium of stallion semen.

In the present study, the purpose was to evaluate the effects of sericin supplementation to semen extender with or without GSH on the frozen/thawed stallion spermatozoa. Therefore, motility parameters, morphology, lipid peroxidation, GPx, SOD, catalase activities, capacitation, DNA and plasma membrane integrity of spermatozoa were also assessed in frozen–thawed stallion semen.

## Materials and Methods

### Material

All chemicals and reagents were obtained from Sigma (St. Louis, MO) and Gibco (Grand Island, NY, USA), respectively, unless otherwise stated in the text.

### Semen collection and processing

Semen was collected (Three times each) from five fertile stallions, 8 to 10 years old, using an artificial vagina (Missouri model, IMV, France). After filtration, the gel-free fraction of the ejaculates was immediately transported to the laboratory for evaluation of spermatozoa quality. Only ejaculates with good wave motion (>60%) were considered for the experiments 28. The samples after dilution in INRA 82 medium (0.5 *L* saline solution: 25 *g* glucose, 1.5 *g* lactose, 1.5 *g* raffinose, 0.25 *g* sodium citrate dihydrate, 0.41 *g* potassium citrate, 50000 *IU* penicillin, 50 *mg* gentamicin, and 0.5 *L* skim milk) containing 2% centrifuged egg yolk (*v/v*) and 20 *mM* HEPES 29, were centrifuged at 600 *g* for 5 *min*. The resulting pellets were re-suspended in freezing medium (INRA 82 medium) containing 2% (*v/v*) egg yolk, 20-*mM* HEPES, and 2.5%, glycerol (*v/v*; pH=6.8) and were then considered for experimental groups.

### Study design

The diluted semen with freezing medium (ratio 1:1) were randomly divided into the following groups: C) without any supplementation, served as control, S) supplemented with 0.25% sericin, G) supplemented with 1.5 *mM* Glutathione, and S+G) supplemented with 0.25% sericin+1.5 *mM* Glutathione. Fifteen ejaculates (three ejaculates from each stallion) after freeze-thaw process were considered for evaluation of the total and progressive motility, lipid peroxidation, DNA and plasma membrane integrity, and enzymatic antioxidant activity of superoxide dismutase, catalase, and glutathione peroxidase.

### Freezing and thawing

Semen freezing was carried out according to Vidament *et al* 30. In brief, the loaded 0.5 *ml* plastic straws with diluted sperm with a final concentration of 2×10^8^ were slowly cooled to 4*°C* within 60 *min*. The straws were horizontally placed in racks 4 *cm* above the surface of Liquid Nitrogen (LN_2_) for 12 *min*, and then directly plunged in LN_2_. Two weeks later, the straws were then thawed in a water bath at 37*°C* for 40 *s*.

### Post thaw sperm analysis

The motility characteristics of sperm including Amplitude of Lateral Head displacement (ALH), Beat Cross Frequency (BCF), curvilinear velocity (VCL), straight line velocity (VSL), average path velocity (VAP), linearity (LIN=VSL/VCL×100), Mean Angular Displacement (MAD), wobble (WOB=VAP/VCL×100), and straightness (STR=VSL/VAP×100) were evaluated after thawing using computer assisted semen analyzers (CASA, Houshm and Fanavar, Iran). The spermatozoa were classified into four classes: class A, fast progressively motile spermatozoa; class B, weak progressively motile spermatozoa; class C, non-progressively motile spermatozoa; and class D, immotile spermatozoa. Both classes of A and B were considered as the percentage of progressive motile spermatozoa.

### Biochemical assays

The frozen-thawed sperm after homogenizing with ultrasonic homogenizer were subjected to the total protein content assessment using the Bio-Rad protein assay kit (Bio-Rad, Hercules, CA) according to the manufacturer instructions.

Lipid peroxidation was determined by measuring the amount of malondialdehyde (MDA) in the samples using the method of Buege and Aust 31. Briefly, the thawed and diluted samples with distilled water, were treated with 2 *ml* of TBA-TCA-HCl reagent (Thiobarbituric acid 0.37%, trichloroacetic acid 15% and 0.25 N HCl) and after coverage with foil were then boiled for 15 *min* in water bath. The samples were then cooled and centrifuged at 3000 *g* for 10 *min* at room temperature. The absorbance of supernatant was measured with a spectrophotometer against reference blank at 532 *nm*.

SOD activity in spermatozoa was assayed according to the method of Kakkar *et al* 32. In brief, 50 *µl* spermatozoa were added to 450 *µl* distilled water, and treated with 2.45 *ml* reaction mixture (Xanthine, Nitroblue tetrazolium; NBT, and phosphate buffer). Subsequently, 50 *µl* of xanthine oxidase (0.1 *mg/ml*) was added to the mixture, and the reaction was terminated by adding 50 *µl* copper dichloride (6 *mM*) every 30 *s* up to 5 *min*. The absorbance was recorded at 560 *nm* and the results were reported as the inhibition rate of NBT reduction.

GPx activity was measured by the method described by Lawrence and Burk 33. Briefly, 20 *µl* of spermatozoa were added to 980 *µl* of the reaction mixture (1 *ml* phosphate buffer, 100 *µl* 20 *mM* sodium azide, 100 *µl* 3 *mM* NADPH, 100 *µl* 10 *mM* EDTA, 100 *µl* 20 *mM* reduced glutathione, and 200 *µl* 2 *U* glutathione reductase) and incubated at 37*°C* for 5 *min*. Ten *µl* of hydrogen peroxide was then added to the above mixture. The change in absorbance at 340 *nm* was monitored against a blank every 30 *s* up to 2 *min*. GPx activity unit is defined as *μmol* of NADPH consumed per *min* per *mg* protein.

The catalase (CAT) activity was assayed as described by Goth 34. In brief, 40 *µl* spermatozoa after dilution in 160 *µl* of potassium phosphate buffer were incubated in 1 *ml* reaction mixture (65 *mM* hydrogen peroxide in 60 *mM* potassium phosphate buffer, pH= 7.4) at 37*°C* for 1 *min*. The reaction was stopped by the addition of 1 *ml* 32.4 *mM* ammonium molybdate solution. The absorbance was measured at 405 *nm* against a blank (1 *ml* reaction mixture, 1 *ml* ammonium molybdate solution and 200 *µl* of potassium phosphate buffer). One unit of CAT was defined as the amount of enzyme catalyzing the degradation of 1 *μmol* hydrogen peroxide per *min*.

### Chromatin structure assay

**Figure 1.**
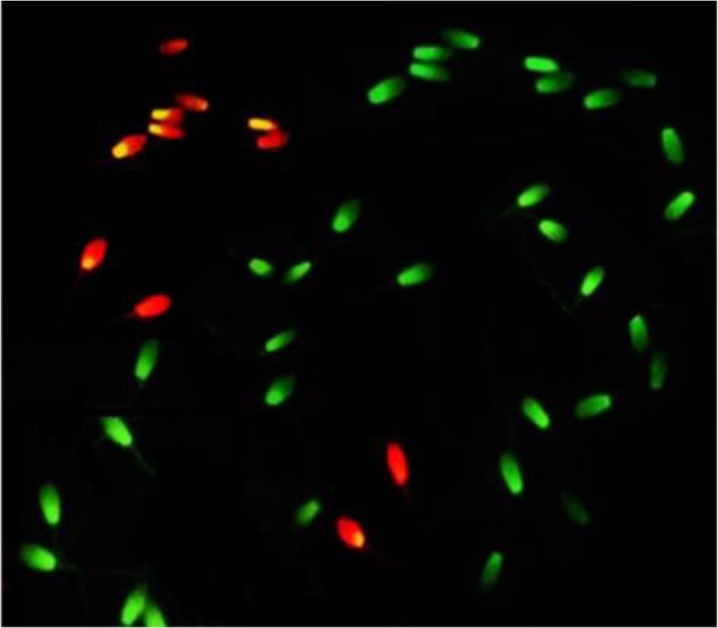
Stallion sperm stained with 1% Acridine Orange (AO). Sperm with normal chromatin structure display green fluorescence and those with an abnormal DNA (Single-strand) shows a yellow to red fluorescence color.

Two smears from each ejaculate were prepared on glass slides and air-dried. The smears after fixation in Carnoy’s solution (3:1 methanol:glacial acetic acid) for 2 *hr* were rinsed with distilled water and then stained with 1% Acridine Orange (AO) for 10 *min* and after washing, covered with a coverslip and immediately sealed with nail polish. All slides were evaluated with an epifluorescent microscope (Olympus BX51, Japan) at 400× magnification. Spermatozoa with normal chromatin structure were stained as green heads, while spermatozoa with abnormal DNA (Single-strand) were colored from yellow to red (Figure 1). A minimum of 200 spermatozoa per slide were evaluated and percentage of spermatozoa with intact chromatin was calculated for each sample.

### Plasma membrane integrity

The Hypo-Osmotic Swelling Test (HOST) was used to evaluate spermatozoa membrane integrity after freeze-thaw process. Briefly, 30 *µl* of each semen sample was added to 200 *ml* of hypo-osmotic solution (25 *mM* sodium citrate dihydrate and 75 *mM* fructose) and incubated at 37*°C* for 30 *min*. Then, the smear was prepared on glass slides and covered with a coverslip. At least 200 spermatozoa were counted under phasecontrast microscope at 400× magnification, and percentage of spermatozoa with coiled tail (Intact membrane) was recorded for each sample.

### Sperm morphology

**Figure 2.**
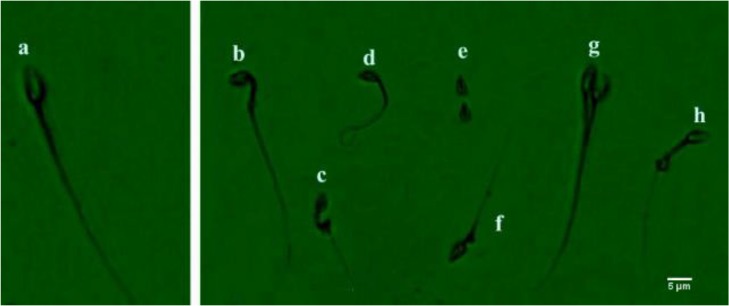
Sperm morphology using Diff-Quik. Sperm with normal. a) and abnormal morphologies (b–h). b) bent midpiece; c) abnormal midpiece; d) bent tail; e) tailless head; f) proximal droplet; g) abnormal head; h) distal droplet.

To evaluate the spermatozoa morphology, samples were stained with Diff-Quick staining. Briefly, from each semen sample, duplicate smears were prepared on glass slides and after fixation, the smears were stained with staining solutions according to the instruction of Diff-Quick staining kit (Avicenna, IRI). The morphology of at least 200 spermatozoa per slide was evaluated under a bright field microscope at 100X magnification and the percentage of normal and abnormal sperm was recorded in each treatment group (Figure 2).

### Capacitation state of spermatozoa

**Figure 3.**
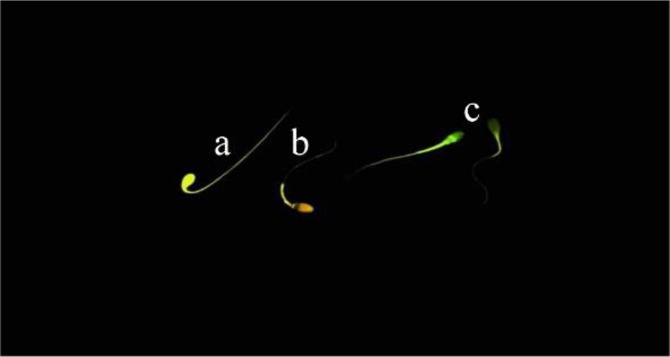
Capacitation state of stallion spermatozoa using chlortetracycline (CTC) staining. a) uncapacitated acrosome-intact spermatozoa (F-pattern); b) capacitated acrosome-intact spermatozoa (B-pattern); c) acrosome reacted spermatozoa (AR-pattern).

The Chlortetracycline (CTC) assay was used to assess the capacitation state of spermatozoa. Briefly, 20 *µl* of CTC solution was laid on a glass slide and thoroughly mixed with 20 *µl* of each semen sample and after fifteen *s*, 5 *µl* of fixative solution was added to the suspension. Afterward, 3 *µl* of antifade solution was added and the slide covered with a coverslip and sealed with nail polish. The slides were stored at 4*°C* in dark and examined at 100× magnification within 2 *hr* of preparation under epifluorescence microscope (Olympus BX51, Japan). Two hundred sperm with duplicate from each sample were assessed according to those described by Schembri *et al* 35. The patterns of staining were considered as follows: F-pattern; green fluorescence over the entire spermatozoa head, uncapacitated sperm with intact acrosome, B-pattern; fluorescence-free band on the post-acrosomal area, capacitated sperm with intact acrosome, and AR-pattern; non-fluorescene over the entire spermatozoa head or except for a thin line of fluorescence on the equatorial area, acrosome-reacted spermatozoa (Figure 3). Spermatozoa that did not fit into any of these described patterns, were not considered for analysis.

### Statistical analysis

Normality and homogeneity of variances were confirmed using Kolmogorov–Smirnov and Levene’s tests, respectively. Statistical analyses were conducted with SPSS 20.0 (SPSS, Chicago, IL, USA). Data were analyzed by one-way ANOVA followed by LSD post hoc test, and differences were considered significant at p< 0.05. Results were presented as mean±SEM.

## Results

### Spermatozoa parameters

As shown in table 1, supplementation of freezing medium with antioxidants had no significant effect on the spermatozoa motility parameters (total motility, progressive motility, VCL, VSL, VAP, MAD, ALH, BCF, LIN, WOB and STR). Among all classes of spermatozoa, only class B (weak progressive motile spermatozoa) was higher in Sericin (S) compared to Glutathione (G) group (p<0.05).

**Table 1.** The effect of supplementation of semen extender with antioxidants on sperm motility parameters in frozen–thawed stallion sperm

**Parameters**	**Control**	**Sericin**	**Glutathione**	**Sericin+Glutathione**
**Motile spermatozoa (%)**	44.74±5.49	49.89±4.06	44.10±5.50	49.30±6.70
**Progressive spermatozoa (%)**	32.53±7.20	38.57±5.50	30.92±7.00	37.48±9.10
**Class A (%)**	19.90±5.70	20.28±6.20	18.43±7.02	23.32±8.50
**Class B (%)**	12.64±1.65 ^ab^	18.29±2.30 ^a^	12.50±0.59 ^b^	13.76±1.99 ^ab^
**Class C (%)**	12.20±2.00	11.31±2.27	13.18±2.00	11.83±2.50
**Class D (%)**	55.27±5.40	50.11±4.06	55.90±5.59	50.70±6.70
**VCL (*µm/s*)**	34.97±5.57	36.34±5.15	34.87±5.70	37.91±7.49
**VSL (*µm/s*)**	17.48±3.98	19.76±4.42	16.73±5.21	22.19±7.01
**VAP (*µm/s*)**	21.77±4.22	24.07±4.54	20.98±5.37	26.5±7.16
**MAD (°)**	10.56±3.99	8.94±2.51	8.74±2.35	8.33±2.55
**ALH (*µm*)**	1.94±0.16	2.05±0.12	1.95±.19	1.97±0.20
**BCF (*Hz*)**	0.31±0.12	0.29±0.10	0.27±0.11	0.32±0.13
**LIN (%)**	32.91±4.38	39.73±4.05	31.44±6.14	38.08±7.80
**WOB (%)**	45.78±4.15	52.97±3.57	44.69±5.98	50.56±7.16
**STR (%)**	48.90±5.19	55.10±3.86	47.96±6.32	51.92±7.62

VCL: Curvilinear velocity, VSL: Straight line velocity, VAP: Average path velocity, MAD: Mean angular displacement, ALH: Amplitude of lateral head displacement, BCF: Beat cross frequency, LIN: Linearity index (LIN= VSL/VCL×100), WOB: Wobble (WOB=VAP/VCL×100), and STR: Straightness index (STR=VSL/VAP×100).

a, b) Different superscripts within the same row demonstrate significant difference (p<0.05).

### Biochemical parameters

The effect of freezing medium supplementation with antioxidants on lipid peroxidation and enzymatic antioxidant activities of thawed stallion semen are shown in table 2. As shown, the level of lipid peroxidation in sericin supplemented group was significantly lower than other treatment groups (p<0.01), though, sericin in combination with glutathione had no such effect. Similarly, CAT and GPx activities of frozen-thawed semen were significantly higher when the extender medium was supplemented only with sericin compared to other groups (p<0.01). Nevertheless, extender supplementation with sericin or other antioxidants could not improve the percentage of SOD inhibition compared to control.

**Table 2.** The effect of supplementation of semen extender with antioxidants on lipid peroxidation and enzymatic antioxidant activity of frozen–thawed stallion semen

**Group**	**CAT (*U/mg*pr)**	**GPx (*U/mg*pr)**	**MDA (*nmol/mg*pr)**	**SOD (% inhibition)**
**Control**	43.95±1.60 ^b^	64.97±2.50 ^b^	29.43±1.24 ^a^	14.49±0.42
**Sericin**	55.22±1.65 ^a^	75.36±2.19 ^a^	23.49±0. 96 ^b^	14.53±0.62
**Glutathione**	46.64±1.21 ^b^	62.28±2 ^b^	30.51±1.12 ^a^	14.58±0.49
**Sericin+Glutathione**	47.75±2.35 ^b^	63.04±3.4 ^b^	29.03±0.83 ^a^	14.22±0.26

CAT: Catalase, GPx: Glutathione peroxidase, MDA: Malondialdehyde, and SOD: Superoxide dismutase.

a, b: Different superscripts within the same column demonstrate significant difference (p<0.01).

### Chromatin structure and plasma membrane integrity

**Figure 4.**
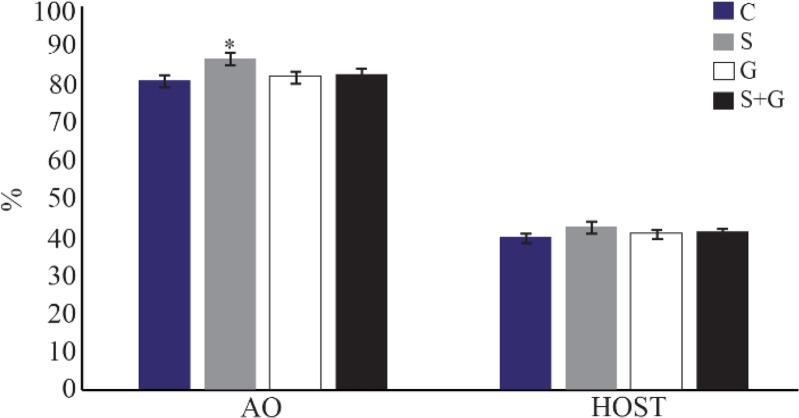
Effect of sericin and glutathione supplementation to the freezing medium of stallion semen on sperm chromatin structure (AO) and its plasma membrane integrity (HOST). AO: acridine orange, HOST: hypo-osmotic swelling test, C: control, S: sericin, G: glutathione, S+G: sericin+ glutathione. Values presented as %± SEM. * p<0.05, compared to other groups.

As shown in figure 4, the percentage of spermatozoa with normal DNA structure was significantly higher in sericin supplemented group compared with other groups (p<0.05). No significant difference was observed among groups regarding plasma membrane integrity detected by hypo-osmotic swelling test (p< 0.05).

### Morphology of spermatozoa

As shown in table 3, the abnormality of sperm heads was lower in the sericin supplemented group (5.20± 0.13) compared to the control (6.20±0.48; p<0.05). The midpieces abnormality was also lower in the S group (1.53±0.08) compared to the other groups (p<0.05). The distal droplet in the G (6.40±0.40) and S+G (6.53± 0.22) groups was lower than the C group (7.40±0.22; p<0.05). There were no significant differences among groups in terms of other abnormalities (p>0.05).

**Table 3.** Sperm morphology of frozen–thawed stallion semen in the presence of antioxidants

**Parameters (%)**	**Control**	**Sericin**	**Glutathione**	**Sericin+Glutathione**
**Normal**	61.87±0.24	61.87±0.29	62.13±0.13	61.67±0.38
**Abnormal heads**	6.20±0.48 ^a^	5.20±0.13 ^b^	5.40±0.24 ^ab^	5.67±0.23 ^ab^
**Abnormal midpieces**	1.93±0.12 ^a^	1.53±0.08 ^b^	2.20±0.13 ^a^	2.20±0.08 ^a^
**Bent midpieces**	6.47±0.27	6.67±0.38	6.47±0.30	6.27±0.19
**Proximal droplet**	7.73±0.06	8.47±0.30	7.60±0.41	8.00±0.31
**Distal droplet**	7.40±0.22 ^a^	6.80±0.22 ^ab^	6.40±0.40 ^b^	6.53±0.22 ^b^
**Bent tails**	6.80±0.38	7.53±0.22	7.47±0.34	7.73±0.26
**Tailless heads**	1.60±0.06	1.93±0.43	2.33±0.33	1.93±0.19

a, b: Different superscripts within the same row demonstrate significant differences (p<0.05).

### Capacitation state of spermatozoa

**Figure 5.**
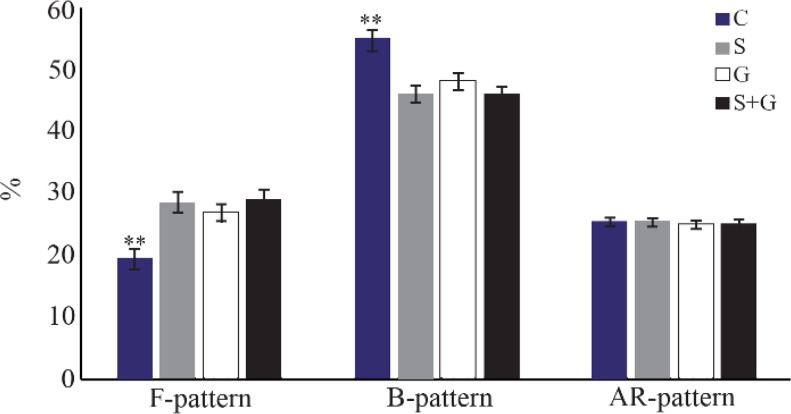
Effect of sericin and glutathione supplementation to the freezing medium of stallion semen on capacitation state of spermatozoa. C: control, S: sericin, G: glutathione, S+G: sericin+ glutathione. Values presented as %±SEM. ** p<0.01, compared to other groups.

As shown in figure 5, the percentage of uncapacitated spermatozoa (F-pattern) in frozen–thawed semen in antioxidants supplemented groups was greater than control (p<0.01). On the other hand, the percentage of capacitated spermatozoa (B-pattern) in control was higher than treatment groups (p<0.01). There was no difference among groups in terms of acrosome-reacted spermatozoa (AR-pattern; p>0.05).

## Discussion

The process of cryopreservation with production of oxidants and free radicals can damage the sperm. In the present study, adding sericin as a natural antioxidant, to INRA82 extender of stallion semen, alone or in combination with glutathione, slightly improved some of the sperm quality parameters. Kumar *et al* evaluated the effect of adding different concentrations of sericin to freezing medium of buffalo semen and concluded that 0.25, 0.5, and 1% of sericin ameliorated the total and progressive spermatozoa motility 36. There are conflicting reports regarding the effects of glutathione on the sperm motility parameters in different species, as some of them have shown positive effects 12,18,34,35 while others have indicated negative effects 17,19,36,37. This difference among the studies may be related to the kind of semen extender, dosage of GSH and/or inter-species differences. A dose-response study perhaps may be helpful to determine the optimal concentration of GSH.

The result of biochemical assay showed that enzymatic antioxidant system (Except for the SOD activity) was improved in the frozen-thawed stallion semen when the extender was supplemented with sericin alone. Moreover, MDA concentration was significantly decreased by adding sericin when compared to the control and other treatment groups. This finding was in agreement with previous study where adding 0.25 and 0.5% sericin to the frozen-thawed buffalo semen had positive effects on GPx activity and LPO amelioration 36.

The GPx and catalase enzymes play an important role in hydrogen peroxide removal and enhancement of GPx and catalase activities, which in turn leads to an increase in antioxidant capacity of the sperm 37. In a study on the skin fibroblast cell line (AH927) treated with hydrogen peroxide, it has been shown that sericin reduces the lactate dehydrogenase and malondialdehyde activities. Similarly, in our study, sericin as a natural antioxidant, could reduce LPO and protect the spermatozoa against oxidative stress. Although the antioxidant properties of sericin have been confirmed by several studies 38,39, its scavenging mechanisms have not been clearly identified. One possible mechanism may be related to the chelating effect of hydroxyl group of serine and threonine that are dominant amino acids (about 40%) in the sericin protein. However, due to the low concentration of these amino acids with thiol group, it is unlikely that this is the reason for sericin antioxidant activity 24.

In the present study, plasma membrane integrity (HOST) was not affected by sericin treatment. It has been demonstrated that SOD, as an enzymatic antioxidant, plays a positive role in the spermatozoa membrane integrity following cryopreservation process. On the other hand, due to physical damage to sperm plasma membranes in the cryopreservation process and SOD leakage 40, the protective effect of SOD on plasma membrane is reduced. Since in our study the sericin had no positive effect on SOD activity, no improvement was observed in sperm membrane integrity.

In the present study, the addition of GSH alone or in combination with sericin could not protect the stallion spermatozoa from free radicals and LPO which was in agreement with Zhandi and Ghadimi study who reported 2 *hr* exposure of miniature Caspian horse semen to GSH which could not improve the plasma membrane integrity 41,42. However, in another study, the addition of 2.5 *mM* GSH to the freezing extender could increase the spermatozoa membrane integrity 43. This discrepancy may be related to the kind of semen extender and/or dosage of GSH.

Stallion sperm DNA integrity was ameliorated in the frozen-thawed semen in the presence of sericin. ROS in a dose-dependent manner is considered as a reason for DNA damage in the stallion spermatozoa. It has been shown that catalase but not superoxide dismutase prevents the DNA damage, which indicates that hydrogen peroxide is the main ROS that leads to DNA damage in stallion spermatozoa 44. Therefore, boosting catalase activity by sericin might be the reason for improvement of the sperm DNA integrity.

Diff-Quick staining showed that the number of spermatozoa with abnormal heads and midpieces in sericin group and spermatozoa with distal droplet in glutathione and sericin+glutathione groups was lower than the one in control group. It has been demonstrated that swelling of the midpiece, mitochondrial enlargement, is a major threat of cryo damage to spermatozoa that leads to the disruption of redox metabolism, promotion of ROS generation and induction of apoptosis 3. It seems sericin keeps mitochondria from cryo damage which subsequently, resulted in reduction of ROS generation.

The sperm capacitation and acrosome-reaction are promoted by cryopreservation, which may have an adverse effect on fertility. However, both G and S+G groups could not ameliorate lipid peroxidation and enzymatic antioxidant activity. The percentage of intact and capacitated spermatozoa in all treated frozen–thawed groups was higher and lower than control, respectively. It has been demonstrated that capacitation is accompanied by an increase in intracellular calcium concentration, ROS generation, cAMP concentration, and elevation of tyrosine phosphorylation. In this context, catalase can suppress the sperm capacitation through inhibition of tyrosine phosphorylation 45. Thus, promotion of catalase activity in the presence of sericin may suppress the capacitation state of the frozen thawed stallion spermatozoa. It seems that GSH has another mechanism in ameliorating the capacitation state of stallion spermatozoa. Previous studies on astrocytes and PC12 cells showed that depleted GSH levels were associated with a rise in intracellular calcium levels 46,47. Accordingly, a possible hypothesis was proposed for the effect of antioxidants on capacitation state of frozen-thawed stallion spermatozoa in treatment groups. In fact, by increasing the amount of GSH in antioxidant treated groups, the amount of intracellular calcium decreases, resulting in a decrease in cAMP and tyrosine phosphorylation. Finally, these events reduce the percentage of capacitated sperm in antioxidant-treated groups.

## Conclusion

In conclusion, this study indicated that supplementation of semen extender with sericin prior to cryopreservation could improve the enzymatic antioxidant activity, DNA integrity, lipid peroxidation, capacitation state, and somehow the morphologic characteristics of the stallion sperm which may be applicable to other species.
